# Reduction of mortality by catheter ablation in real-world atrial fibrillation patients with heart failure

**DOI:** 10.1038/s41598-021-84256-z

**Published:** 2021-02-25

**Authors:** Pil-Sung Yang, Daehoon Kim, Jung-Hoon Sung, Eunsun Jang, Hee Tae Yu, Tae-Hoon Kim, Jae-Sun Uhm, Jong-Youn Kim, Hui-Nam Pak, Moon-Hyoung Lee, Boyoung Joung

**Affiliations:** 1 Department of Cardiology, CHA Bundang Medical Center, CHA University, Seongnam, Republic of Korea; 2 Division of Cardiology, Department of Internal Medicine, Yonsei University College of Medicine, 50-1 Yonsei-ro, Seodaemun-gu, Seoul, 03722 Republic of Korea

**Keywords:** Atrial fibrillation, Heart failure, Outcomes research

## Abstract

Whether catheter ablation for atrial fibrillation (AF) improves survival and affects other outcomes in real-world heart failure (HF) patients is unclear. This study aimed to evaluate whether ablation reduces death, and other outcomes in real-world AF patients with HF. Among 834,735 patients with AF from 2006 to 2015 in the Korean National Health Insurance Service database, 3173 HF patients underwent AF ablation. Propensity score weighting was used to correct for differences between the groups. During median 54 months follow-up, the risk of all-cause death in ablated patients was less than half of that in patients with medical therapy (2.8 vs. 6.2 per 100 person-years; hazard ratio [HR] 0.42, 95% confidence interval [CI] 0.27–0.65, *p* < 0.001). Ablation was related with lower risk of cardiovascular death (HR 0.38, 95% CI 0.32–0.62, *p* < 0.001), HF admission (HR 0.39, 95% CI 0.33–0.46, *p* < 0.001) and stroke/systemic embolism (HR 0.44, 95% CI 0.37–0.53, *p* < 0.001). In subgroup analysis, the risk of all-cause death was reduced in most subgroups except in the elderly (≥ 75 years) and strictly anticoagulated patients. Ablation may be associated with reduced risk of all-cause death and cardiovascular death in real-world AF patients with HF, supporting the role of AF ablation in patients with HF.

## Introduction

Atrial fibrillation (AF) and heart failure are important cardiac conditions associated with the patient's morbidity and mortality^1,2^. The two conditions often coexist and can promote each other. Up to 30% of patients with heart failure have AF^3–5^. AF in patients with heart failure is associated with increased hospitalization, the burden on the health care system, stroke, and mortality^6,7^. Loss of atrial contraction, irregular and rapid ventricular rates in AF can lead to left ventricular dysfunction and decreased cardiac output^8,9^, and these features of AF may be at least partially contributed to poor prognosis of heart failure patients.

Catheter ablation for AF is more effective than antiarrhythmic drugs (AADs) in reducing AF recurrences; AF catheter ablation also extends the duration of the sinus rhythm and improves the patient's quality of life^10,11^. Several observational studies have shown that maintaining sinus rhythm by AF catheter ablation in heart failure patients can significantly improve cardiac function^12–14^. Recently, a trial evaluating ablation compared with medical therapy in symptomatic patients with AF and heart failure provided evidence suggesting that successful ablation may extend survival^15^. However, because only 13.2% of the screened heart failure patients were enrolled in randomization in the study, the effect of AF ablation in heart failure patients is still controversial. In the CABANA (Catheter Ablation vs. Antiarrhythmic Drug Therapy for Atrial Fibrillation) trial, the recently performed randomized controlled trial on the effects of AF catheter ablation^16^, AF catheter ablation did not significantly reduce the primary endpoint (a composite of death, disabling stroke, serious bleeding, or cardiac arrest) compared to medical treatment.

Our study aimed to determine whether AF catheter ablation in heart failure patients can reduce the risk of all-cause death, cardiovascular death, and other cardiac events in a real-world nationwide cohort.

## Methods

This study was based on the national health claims database (NHIS-2016-4-009) established by the National Health Insurance Service (NHIS) of Korea. The NHIS is the single insurer managed by the Korean government. The majority (97.1%) of Korean citizens are mandatory subscribers to the NHIS, and the remaining 3% of the population is under the Medical Aid program. As the NHIS database contains the information of Medical Aid users, the database can be considered to be representative of the entire Korean population^3–5^. All pertinent data including patients’ sociodemographic information, use of inpatient and outpatient services, pharmacy-dispensing claims, and mortality can be accessed through this database. The NHIS also runs a regular health check-up program for all citizens. NHIS subscribers are recommended to undergo check-ups at least biennially, and the check-up includes blood tests, chest X-ray, physical examinations, and questionnaires for medical history.

The present study was approved by the Institutional Review Board of Yonsei University Health System (4-2016-0179). The board waived the condition of obtaining informed consent for study participation. All methods included in this study were carried out in accordance with relevant guidelines and regulations.

### Study population

From January 1, 2006, to December 31, 2015, 834,735 adult patients (18 years old) newly diagnosed with AF were identified in the Korean NHIS database covering a population of 51.5 million. AF was confirmed by the diagnostic code (International Classification of Disease 10th revision [ICD-10] code: I48). Only patients who were diagnosed during hospitalization or diagnosed at least two times in an outpatient clinic were confirmed as AF to ensure the accuracy of the diagnosis. The accuracy of this definition has already been validated in previous studies using the Korean NHIS data. A positive predictive value was 94.1%^3–5,17–19^. From newly diagnosed AF patients, patients treated with AF catheter ablation or medical therapy (AADs or rate control drugs prescribed for at least 90 days within one year of enrollment) were included in the study population. Catheter ablation for AF was identified using the corresponding Korean NHIS procedure codes for AF catheter ablation (M6542 or M6547) with an admission diagnosis of AF.

**Figure 1:**
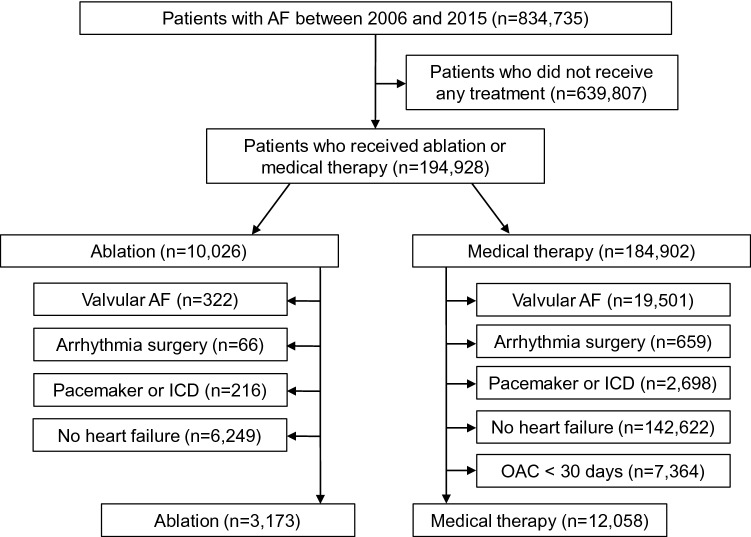
Flowchart of the enrollment and analysis of the study population. AF, atrial fibrillation; ICD, implantable cardioverter-defibrillator; OAC, oral anticoagulant.

The time at risk was counted from the index date of the first AF treatment for both patients with AF ablation and those with medical therapy. The time at risk in patients who underwent AF ablation without prior medical treatment was counted from the date of the first AF ablation. The effect of AF ablation was analyzed as a time-varying exposure. The exclusion criteria were those with mitral stenosis, a history of mitral valve replacement, surgical AF ablation (Maze surgery), or implantation of cardiac implantable electronic device, and those without heart failure history. Patients who had oral anticoagulants less than 30 days during the same period were additionally excluded from the medical therapy group. After exclusions, 3173 ablated patients and 12,058 medically treated patients remained for analysis (Fig. 1).

### Covariates

Baseline comorbidities were defined based on inpatient and outpatient clinic diagnosis and prescription drugs prior to the index date. Similar to previous studies using Korean NHIS data, patients diagnosed in admission or confirmed at least twice in an outpatient clinic were considered to have a comorbid (Supplementary Table S1)^3–5,17–19^. The patient's economic status was determined on the basis of the relative economic level classified into 10 tiers according to their health insurance premium of the index year. The use of medication was identified based on prescription claims in the NHIS database within 90 days prior to the index date.

### Clinical outcome events and assessments

To assess clinical outcomes, patients were followed until the end of the study period (December 31, 2016) or until death. All-cause death, cardiovascular death, and sudden cardiac death were evaluated. Vital status and the date of death were identified from the data registered in the National Population Registry of the Korea National Statistical Office, based on death certificates and the unique personal identification number^3–5,17–19^. Since the NHIS and National Statistical Office are national organizations covering all Korean citizens, this approach allows complete confirmation of death events.

The definitions of clinical outcomes are presented in Supplementary Table S1. Ischemic stroke was identified by discharge diagnosis (ICD-10 codes: I63, I64) with concomitant brain imaging studies. The accuracy of this definition for ischemic stroke has been validated in previous studies using the Korean NHIS data^3–5,17–19^. It must be noted that only the first event of each outcome was considered in the study when there are multiple events.

To indirectly assess the relationship between successful rhythm control by ablation and outcomes, we considered “Cardioversion or repeated ablation” as an indicator of AF recurrence after ablation. We also performed a validation study of this definition for detecting AF recurrence (Supplement Figure S1).

### Statistical methods

Baseline characteristics between patients with AF ablation and those with medical therapy were compared using Student’s t-test and chi-square test. A propensity score, the probability of receiving AF ablation, was estimated using logistic regression based on socio-demographics, concomitant diseases, drug use, and duration of AF. (All variables in Table 1 were used). On the basis of the calculated propensity score, inverse probability of treatment weighting was used to balance the differences in baseline characteristics between patients with AF ablation and those with medical therapy. The balance between the two groups was assessed with standardized differences of all baseline covariates, using 0.1 as the threshold representing the imbalance.

**Table 1:** Baseline characteristics before and after propensity score weighting.

	Ablation (N = 3173)	Medical therapy (N = 12,058)	SMD (%)	Ablation (N = 3173)	Medical therapy (N = 12,058)	SMD (%)
**Demographic**						
Age, years	60 (53, 67)	69 (62, 76)	82.2	66 (58, 74)	66 (58, 74)	0.9
< 65 years	67.4%	33.4%	72.2	42.8%	44.2%	2.8
65–75 yearswprk dl	26.3%	32.7%	14.1	29.9%	30.4%	1.0
≥ 75 years	4.8%	30.3%	71.0	24.0%	22.5%	3.5
Male	71.5%	59.1%	26.3	64.5%	62.7%	3.7
High income status	51.7%	42.1%	19.2	49.2%	44.3%	9.8
AF duration, months	34.9 (11.9, 66.8)	23.6 (4.6, 46.0)	29.6	20.1 (5.2, 49.5)	23.4 (3.9, 48.7)	5.1
**Risk scores**						
CHA_2_DS_2_-VASc score	3.0 (2.0, 5.0)	5.0 (3.0, 6.0)	73.0	4.0 (3.0, 6.0)	4.0 (3.0, 6.0)	2.9
mHAS-BLED score*	3.0 (2.0, 4.0)	3.0 (2.0, 4.0)	36.3	1.4 (0.8, 2.7)	3.0 (2.1, 3.9)	5.3
Charlson comorbidity index	1.8 (0.0, 4.8)	3.2 (0.0, 8.8)	38.7	5.0 (3.0, 7.0)	5.0 (3.0, 7.0)	1.1
Hospital frailty risk score	4.0 (3.0, 6.0)	5.0 (3.0, 8.0)	62.6	2.9 (0.7, 6.3)	2.3 (0.0, 7.1)	9.0
**Comorbidities**						
Heart failure	100.0%	100.0%	< 0.001	100.0%	100.0%	< 0.1
Hypertension	91.3%	94.7%	13.4	93.9%	92.7%	4.6
Diabetes	17.8%	31.1%	31.3	27.5%	27.0%	1.1
Dyslipidemia	88.5%	80.0%	23.6	83.4%	81.5%	5.0
Ischemic stroke	19.9%	36.1%	36.8	32.9%	30.7%	4.7
TIA	10.1%	11.1%	3.4	10.0%	10.7%	2.5
Hemorrhagic stroke	1.5%	3.4%	12.7	4.8%	2.8%	10.2
Myocardial infarction	13.6%	21.0%	19.7	17.3%	18.5%	3.1
Peripheral arterial disease	14.2%	18.0%	10.3	15.1%	16.7%	4.3
Chronic kidney disease	5.9%	10.1%	15.4	11.2%	8.8%	8.1
End stage renal disease	0.9%	1.5%	5.7	1.4%	1.3%	1.0
Proteinuria	5.9%	6.6%	2.9	6.5%	6.4%	0.6
Hyperthyroidism	23.2%	17.4%	14.5	17.9%	18.4%	1.3
Hypothyroidism	20.6%	14.6%	15.6	13.9%	15.5%	4.6
Malignancy	21.9%	22.8%	2.0	18.9%	22.1%	7.9
COPD	26.2%	38.5%	26.7	36.9%	34.5%	5.1
Liver disease	49.4%	43.4%	12.0	43.7%	44.6%	1.8
Hypertrophic cardiomyopathy	3.3%	4.0%	3.7	3.9%	3.8%	0.7
History of bleeding	33.6%	33.7%	0.3	35.9%	33.4%	5.4
Osteoporosis	20.9%	31.1%	23.4	26.0%	27.9%	4.4
Sleep apnea	2.1%	0.5%	14.4	1.3%	0.9%	3.8
Heart failure admission	7.5%	27.2%	54.0	22.3%	22.3%	< 0.1
**Medication (treatment)**	0.0%	0.0%		0.0%	0.0%	
OAC	68.2%	76.6%	18.9	63.3%	68.9%	12.0
Antiplatelet agents	79.8%	70.6%	21.4	70.6%	71.5%	2.0
ACE-inhibitor/ARB	64.2%	74.4%	22.2	72.5%	70.2%	5.0
Diuretics	53.7%	76.7%	49.6	70.5%	69.3%	2.7
K sparing diuretics	17.5%	34.8%	40.3	29.6%	29.2%	1.0
Statin	46.9%	43.1%	7.6	45.2%	43.1%	4.3
Beta blocker	77.0%	65.3%	25.9	67.1%	66.7%	0.8
Dihydropyridine CCB	34.7%	42.5%	16.1	43.3%	40.0%	6.7
Nondihydropyridine CCB	27.2%	20.1%	16.6	20.9%	21.1%	0.6
Digoxin	23.5%	45.0%	46.6	37.6%	38.7%	2.3

Values are presented as median (Q1, Q3, quartiles [25th and 75th percentiles]) or %. *Modified HAS-BLED = hypertension, 1 point: > 65 years old, 1 point: stroke history, 1 point: bleeding history or predisposition, 1 point: liable international normalized ratio, not assessed: ethanol or drug abuse, 1 point: drug predisposing to bleeding, 1 point.

*ACE* angiotensin converting enzyme, *AF* atrial fibrillation, *ARB* angiotensin II receptor blocker, *CCB* calcium channel blocker, *COPD* chronic obstructive pulmonary disease, *OAC* oral anticoagulant, *SMD* standardized mean difference, *TIA* transient ischemic attack.

Weighted incidence rates were calculated as the weighted number of clinical events during the follow-up period divided by person-years at risk. The 95% confidence intervals (CI) of incidence rates were estimated by exact Poisson distributions. A weighted log-rank test was used to compare the incidence of mortality and weighted failure curves were plotted. Comparisons between the ablation group and the medical therapy group were analyzed using Cox proportional hazards regressions. The Fine and Gray method was used to regard death as a competing risk when evaluated non-fatal outcomes (i.e. heart failure and stroke/systemic embolism (SE) when evaluated separately)^20^. The proportional hazards assumption was tested based on Schoenfeld residuals^21^.

A two-sided *p* values of < 0.05 were considered significant. Data processing and management were performed using SAS version 9.3 (SAS Institute, Cary, NC, USA). Statistical analyses were conducted using R software (version 3.6.1, R Core Team, 2019, Vienna, Austria)^22^.

### Sensitivity analyses

First, we conducted subgroup analyses for all-cause death and cardiovascular death stratified by age, sex, heart failure, hypertension, diabetes, vascular disease, CHA_2_DS_2_-VASc score, history of ischemic stroke/transient ischemic attack (TIA), cardioversion, and anticoagulation. Second, instead of using propensity score weighting, we performed an analysis using one-to-one propensity score matching between groups to balance the differences in baseline characteristics. Third, we performed a stratified analysis according to whether medically treated patients were treated with AADs or only with rate control drugs. Fourth, we compared heart failure patients who underwent AF ablation and those who did not have a history of AF. Fifth, we performed “falsification analysis” to determine whether AF ablation was associated with the risks of falsification endpoints such as urinary tract infections, Varicella-zoster, and fall accidents, which should not be associated with AF ablation^23^.

## Results

### Differences between ablated and the non-ablated patients

Patients treated with AF catheter ablation had more men, healthier, and higher incomes than those who were medically treated (Table 1). Compared with non-ablated patients, patients receiving ablation were on average 10 years younger and had fewer comorbidities. After propensity score weighting, no significant differences in baseline characteristics were observed between the two groups (Table 1). In multivariable analysis, patients with younger age, higher income, and fewer comorbidities (especially without diabetes, ischemic stroke/TIA, myocardial infarction, and peripheral artery disease) were more likely to undergo AF ablation (Supplementary Table S2).

Patients who underwent AF ablation were younger and healthier than AAD treated (Supplementary Table S3) and rate control patients (Supplementary Table S4). After propensity score weighting, no significant differences in baseline characteristics were observed between the groups.

### Reduced all-cause and cardiovascular death in ablated patients

**Figure 2:**
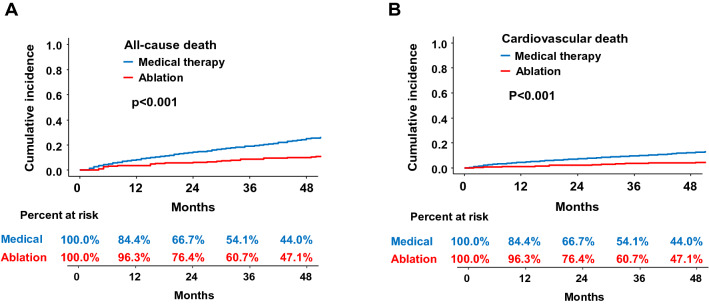
Weighted cumulative incidence curves of (**A**) all-cause death and (**B**) cardiovascular death for ablated and medical therapy patients. Figure prepared in R software (version 3.6.1, R Core Team, 2019, Vienna, Austria)^22^.

**Figure 3:**
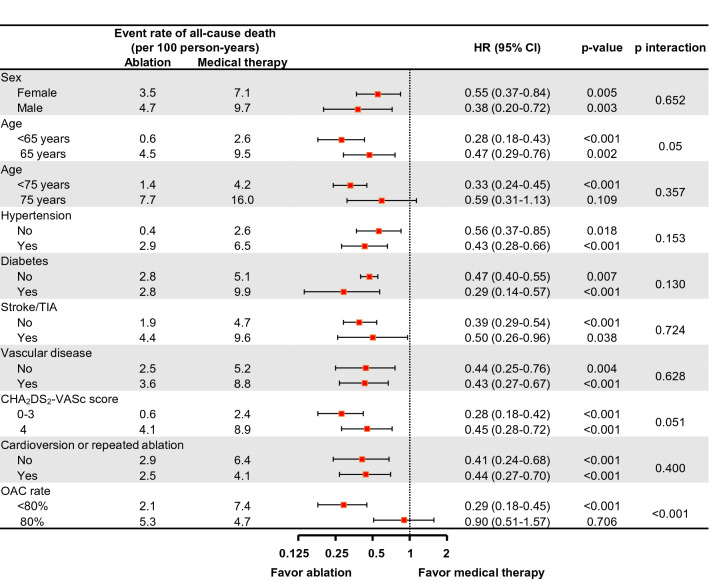
Subgroup analyses of the risk of all-cause death. HR: hazard ratio, TIA: transient ischemic attack, OAC: oral anticoagulant.

In propensity score-weighted patients, 394 and 3940 all-cause deaths occurred during the median 54 (interquartile ranges: 19, 80) months follow-up period, and weighted annualized rates of all-cause death were 2.8 and 6.2 per 100 person-years in the ablated and medical therapy group, respectively (*p* < 0.001) (Table 2). The cumulative incidence of all-cause death in patients with AF ablation was significantly lower than those with medical therapy (*p* < 0.001, Fig. 2A). After fully adjusting the available clinical parameters, the risk of all-cause death was reduced by 58% in ablated patients compared to patients receiving medical therapy (hazard ratio [HR] 0.42, 95% CI 0.27–0.65, *p* < 0.001) (Table 2). The risk of all-cause mortality was reduced in patients with AF ablation compared to those treated with AAD (HR 0.49, 95% CI 0.34–0.71, *p* < 0.001) and rate control only (HR 0.39, 95% CI 0.27–0.57, *p* < 0.001) (Table 2). Subgroup analyses found a reduction in the risk of all-cause death in most subgroups, except for the elderly (≥ 75 years) and optimally anticoagulated (proportion of days covered by anticoagulant ≥ 80%) patients (Fig. 3).

**Table 2:** Risk of clinical outcomes in propensity score-weighted patients stratified by treatment.

	Number of events	Person years	Event rate (100 person-years)	Number of events	Person years	Event rate (100 person-years)	Absolute reduction in event rate (95% CI)	Hazard ratio (95% CI) *	*p* value
**Ablation vs. medical therapy**									
	Medical Therapy (N = 12,058)			Ablation (N = 3173)					
All-cause death	3940	63,486	6.2	394	14,155	2.8	3.4 (3.0–3.9)	0.42 (0.27–0.65)	< 0.001
Cardiovascular death	1912	63,486	3.0	174	14,155	1.2	1.8 (1.5–2.1)	0.38 (0.22–0.62)	< 0.001
Heart failure	2540	54,200	4.7	273	12,937	2.1	2.6 (2.2–3.0)	0.39 (0.33–0.46)	< 0.001
Stroke/SE	2057	56,534	3.6	301	13,130	2.3	1.3 (1.0–1.7)	0.44 (0.37–0.53)	< 0.001
Sudden cardiac death	601	62,868	1.0	65	14,064	0.5	0.5 (0.3–0.7)	0.47 (0.19–1.18)	0.108
**Ablation vs. AAD treated**									
	AAD treated (N = 7976)			Ablation (N = 3173)					
All-cause death	2047	42,158	4.9	345	14,352	2.4	2.5 (2.1–2.8)	0.49 (0.34–0.71)	< 0.001
Cardiovascular death	977	42,158	2.3	153	14,352	1.1	1.3 (1.0–1.5)	0.43 (0.27–0.68)	< 0.001
Heart failure	1574	36,520	4.3	256	13,171	1.9	2.4 (2.0–2.7)	0.35 (0.30–0.42)	< 0.001
Stroke/SE	1222	38,261	3.2	281	13,370	2.1	1.1 (0.8–1.4)	0.45 (0.37–0.54)	0.004
Sudden cardiac death	340	41,716	0.8	58	14,258	0.4	0.4 (0.2–0.6)	0.49 (0.22–1.08)	0.078
**Ablation vs. rate control only**									
	Rate control only (N = 4082)			Ablation (N = 3173)					
All-cause death	1916	30,188	6.3	342	14,415	2.4	4.0 (3.5–4.4)	0.39 (0.27–0.57)	< 0.001
Cardiovascular death	933	30,188	3.1	156	14,415	1.1	2.0 (1.7–2.3)	0.35 (0.22–0.56)	< 0.001
Heart failure	1180	26,143	4.5	253	13,279	1.9	2.6 (2.2–3.0)	0.36 (0.30–0.44)	< 0.001
Stroke/SE	1055	26,492	4.0	286	13,415	2.1	1.9 (1.5–2.2)	0.37 (0.30–0.45)	< 0.001
Sudden cardiac death	289	29,896	1.0	53	14,329	0.4	0.6 (0.4–0.8)	0.43 (0.21–0.88)	0.020

*Adjusted for age, sex, income, AF duration, CHA_2_DS_2_-VASc score, modified HAS-BLED score, hospital frailty risk score, Charlson comorbidity index, hypertension, diabetes, ischemic stroke/TIA, myocardial infarction, peripheral arterial disease, hypertrophic cardiomyopathy, chronic kidney disease, end stage renal disease, liver disease, malignancy, hyperthyroidism, hypothyroidism, venous thromboembolism, COPD, intracranial bleeding, previous cardioversion, history of bleeding, baseline use of warfarin, non-vitamin K antagonist oral anticoagulant, aspirin, clopidogrel, beta-blocker, ACE-inhibitor/ARB, dihydropyridine/nondihydropyridine CCB, statin, diuretics, digoxin, and OAC coverage rate of time at risk.

*AAD* antiarrhythmic drug, *CI* confidence interval, *SE* systemic embolism. Other abbreviations are same as Table 1.

**Figure 4:**
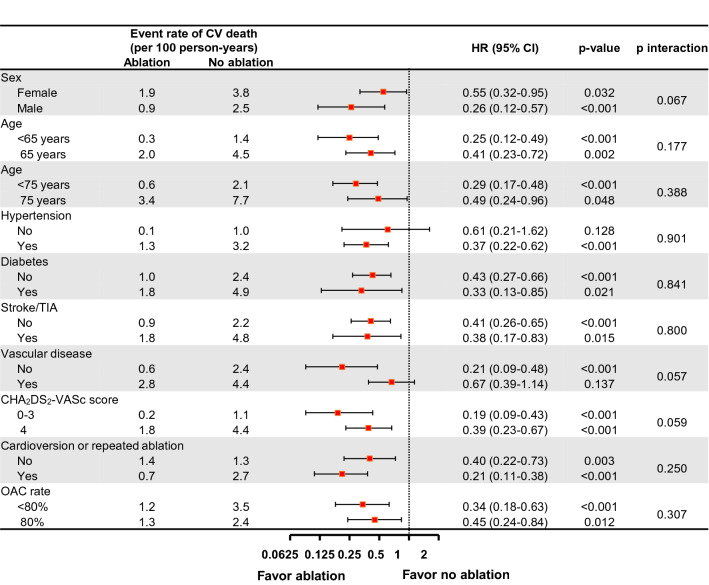
Subgroup analyses of the risk of cardiovascular death. HR, hazard ratio, TIA, transient ischemic attack, OAC, oral anticoagulant.

The risk of cardiovascular death was 62% lower (1.2 and 3.0 per 100 person-years, respectively; HR 0.38, 95% CI 0.32–0.62, *p* < 0.001), and the cumulative incidence of cardiovascular death was significantly lower in patients with AF ablation compared to those with medical therapy (*p* < 0.001) (Table 2, Fig. 2B). The risk of cardiovascular mortality was also reduced in patients with AF ablation compared to those treated with AAD (HR 0.43, 95% CI 0.27–0.68, *p* < 0.001) and rate control only (HR 0.35, 95% CI 0.22–0.56, *p* < 0.001) (Table 2). Subgroup analyses about the risk of cardiovascular death showed that it was reduced in most subgroups except in heart failure patients without hypertension and those with vascular disease (Fig. 4).

### Reduced heart failure admission and Stroke/SE in ablated patients

**Figure 5:**
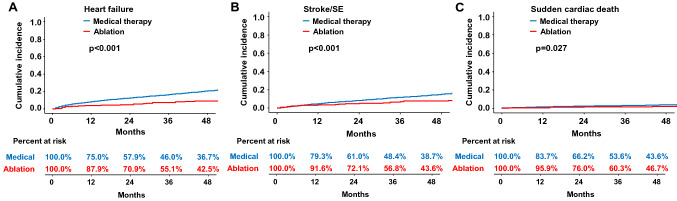
Weighted cumulative incidence curves of (**A**) heart failure, (**B**) ischemic stroke/SE, and (**C**) sudden cardiac death for patients with ablation or medical therapy. SE, systemic embolism. Figure prepared in R software (version 3.6.1, R Core Team, 2019, Vienna, Austria)^22^.

AF ablation in heart failure patients was related to lower incidence and risk of heart failure admission (2.1 and 4.7 per 100 person-years, respectively; HR 0.39, 95% CI 0.33–0.46, *p* < 0.001) and stroke/SE (2.3 and 3.6 per 100 person-years, respectively; HR 0.44, 95% CI 0.37–0.53, *p* < 0.001) compared to the medical therapy (Table 2). The cumulative incidence of heart failure admission (*p* < 0.001, Fig. 5A) and stroke/SE (*p* < 0.001, Fig. 5B) in patients with AF ablation was significantly lower than those with medical therapy.

The risk of heart failure admission in patients with AF ablation was lower than those treated with AAD (HR 0.35, 95% CI 0.30–0.42, *p* < 0.001) and rate control only (HR 0.36, 95% CI 0.30–0.44, *p* < 0.001). AF ablation was also associated with a lower risk of ischemic stroke/SE compared to AAD treatment (HR 0.45, 95% CI 0.37–0.54, *p* < 0.001) and rate control only (HR 0.37, 95% CI 0.30–0.45, *p* < 0.001) (Table 2).

However, the risk of sudden cardiac death of ablated patients was lower than rate control only patients, but not medical therapy or AAD treated patients (Table 2, Fig. 5C).

### Sensitivity analyses

First, the results using one-to-one propensity score matching (instead of propensity score weighting) were consistent with the primary results. HR for all-cause death when AF ablation was performed was 0.38 (95% CI 0.31–0.47, *p* < 0.001) compared to medical therapy, 0.41 (95% CI 0.34–0.51, *p* < 0.001) compared to AAD treatment, and 0.59 (95% CI 0.45–0.77, *p* < 0.001) compared to rate control only. In addition, AF catheter ablation was associated with a lower risk of cardiovascular death, admission for heart failure, and ischemic stroke/SE compared to medical therapy, AAD treatment, and rate control in 1:1 propensity score matching analysis (Supplementary Table S5). Second, a comparison of the group of patients with heart failure who underwent AF ablation and the contemporary matched group of heart failure patients without a history of AF is shown in Supplementary Table S6. Compared with propensity score-weighted heart failure patients without a history of AF, the risks of all-cause mortality, admission for heart failure, and ischemic stroke/SE were not significantly higher in heart failure patients who underwent AF ablation. Third, AF ablation had no significant relationship with any of the falsification endpoints (Supplementary Table S7).

## Discussion

The main finding of this study was that in real-world data, ablation for AF in patients with heart failure was associated with a significantly lower risk of mortality than medical therapy. Second, AF patients with heart failure who underwent ablation had a lower risk of hospitalization for heart failure and stroke/SE than the non-ablated matched patients. However, ablation was not associated with the reduced risk of death in the elderly (≥ 75 years) and optimally anticoagulated patients. This finding supports the beneficial effect of AF catheter ablation in real-world AF patients with heart failure.

### The effect of ablation on mortality and heart failure admission

Complication rates related to the catheter ablation procedure may be higher in patients with heart failure compared with general cohorts of patients undergoing an AF ablation. In the CASTLE-AF (Catheter Ablation versus Standard Conventional Therapy in Patients with Left Ventricular Dysfunction and Atrial Fibrillation) study, procedure-related complications or serious adverse events occurred in 7.8%^15^. In the meta-analysis of patients with heart failure, the peri-procedural major complication rate of AF catheter ablation was 6.3%^24^, while the complication rate in a contemporary cohort of general patients undergoing AF ablation was 2.3%^25^. However, consistently in previous studies of heart failure patients, AF catheter ablation improved left ventricular systolic function and reduced adverse outcomes including readmission due to heart failure^12–14^. A randomized control study, the CASTLE-AF trial, showed that catheter ablation for AF in patients with heart failure was associated with a significantly lower rate of a composite endpoint of death from any cause or hospitalization for worsening heart failure than medical therapy. Ablation lowered the risk of death with an HR of 0.53 (95% CI 0.32–0.86, *p* = 0.01)^15^. In the current study, the risk reduction of death in the ablated group compared to the non-ablated group was very similar to the improvement in outcome for all-cause mortality in the CASTLE-AF trial.

In contrast to the current study and the CASTLE-AF trial, the AMICA (the Atrial Fibrillation Management in Congestive Heart Failure With Ablation) trial, a randomized controlled trial published in 2019, failed to show a significant benefit of catheter ablation over best medical therapy^26^. However, the AMICA trial included only patients with persistent/longstanding persistent AF and heart failure with severely reduced left ventricular ejection fraction (LVEF) less than 35%. Our study included not only persistent AF patients but also paroxysmal AF patients. Heart failure patients with preserved LVEF also might be included because heart failure was defined through the diagnosis code of the administrative database. The limited benefit of catheter ablation in the AMICA trial can be explained because study patients were generally sicker and with more advanced heart failure compared with the patient in our study. Ablation therapy might have limited benefit over medical treatment in patients with seriously advanced heart failure despite achieving a lower AF burden. It should be important to select the patient carefully to perform catheter ablation to maximize the benefit of catheter ablation.

In the subgroup analysis of this study, all-cause mortality was not significantly reduced by ablation in the elderly population and those with strict anticoagulation. Attenuation of the benefits by ablation in the elderly population can also be found in the subgroup analysis of CASTLE-AF and CABANA trial^15,16^. The more advanced left atrial remodeling of older patients and the consequent decrease in AF ablation efficiency may be the cause, but this remains unproven. Anticoagulation therapy (vitamin K antagonists) has been shown to reduce overall mortality in individuals with AF compared with placebo when all studies are considered together (relative risk, 0.74; 95% CI, 0.57–0.97)^27^. No significant reduction of the outcome by ablation in patients with strict anticoagulation emphasizes that effective anticoagulation is the most important to reduce mortality in AF patients with heart failure. Catheter ablation for maintaining sinus rhythm is the next important step. The current study suggests that ablation might be related to the reduction of cardiovascular mortality in real-world AF patients with heart failure.

### The effect of ablation on other outcomes

Ablation also reduced the risk of heart failure hospitalization in the CASTLE-AF trial and this study^15^. This result highlights the importance of preserving or restoring the atrial contribution to cardiac hemodynamics, as the AF catheter ablation rhythm control strategy provides additional benefits over simple control of rapid ventricular rate in patients with heart failure in AF.

The number of events of ischemic stroke was too small to have enough power to prove the benefit of ablation in the prevention of ischemic stroke in the CASTLE-AF study^15^. Several non-randomized observational studies have reported positive outcomes, including reduced incidence of ischemic stroke and reduced mortality in patients with AF ablation^28–31^. But, these favorable studies of reducing stroke after ablation have not been performed in specific patients with AF and heart failure. In the current study of AF patients with heart failure, the risks of ischemic stroke/SE was lower in patients who underwent AF ablation than those with medical therapy. AF and heart failure form a vicious circle with each other. Concomitant AF and heart failure synergistically increase the risk of stroke. Because ablation is an effective treatment for rhythm control that breaks the vicious cycle between AF and heart failure, it can be beneficial in stroke prevention of patients with AF and heart failure.

### Study limitations

There are several limitations to this study. First, because the administrative database is used, it might be susceptible to errors caused by inaccuracies in coding for diagnosis. To minimize these errors, we applied definitions already validated in previous studies using the Korean NHIS data^3–5^. Second, a retrospective registry study like ours cannot establish a causal relationship, only the association can be reported. The propensity score weighting was used to match the two groups as closely as possible, but unknown confounding factors cannot be resolved. Third, because information on the degree of left ventricular function was unavailable, the severity of heart failure was not evaluated. Because the healthier people are more likely to receive ablation as the Supplement Table S2 shows, the heart failure severity could be different between the ablated group and the medical therapy group, which might make bias in the results. Our comparisons between ablation and medical therapy should be interpreted carefully. Fourth, Some study patients had a history of hyperthyroidism, hypothyroidism, malignancy, chronic obstructive pulmonary disease, or liver disease that may affect outcomes. Therefore, there may be concerns that the benefit of ablation in AF patients with heart failure may only be applied to a specific subgroup. Fifth, the utilization rate of oral anticoagulants in this study was lower than in other studies. It is well known that the use of oral anticoagulants in Asia–Pacific countries is low compared to other regions^5,32^. The utilization rate of oral anticoagulants has improved with the introduction of non-vitamin K antagonist oral anticoagulants (NOACs). However, in Korea, the use of NOACs was increased from 2015 as NOACs were fully reimbursed by the national insurance system. The inclusion period of our study was from 2005 to 2015, before NOAC was fully used. So oral anticoagulants were underused in this study population and a large proportion of oral anticoagulants was warfarin. The underuse of oral anticoagulants and a large proportion of warfarin among oral anticoagulants may have influenced the results of this study. Sixth, the dose and label adherence of NOACs was not evaluated. And we also did not have access to information on time-in-therapeutic range in patients using warfarin. Lack of information about the quality of oral anticoagulants treatment might interfere with results, especially the outcome of ischemic stroke and systemic embolism. Finally, to indirectly assess the relationship between successful rhythm control by ablation and all-cause and cardiovascular death, subgroup analysis was performed according to “Cardioversion or repeated ablation” as an indicator of AF recurrence after ablation. However, the exact relationships between the primary outcome or mortality and rhythm control statuses such as sinus rhythm maintenance or AF burden were not evaluated. Moreover, since there is no continuous rhythm monitoring data, the subclinical or asymptomatic AF recurrence can not be assessed.

## Conclusion

Ablation may be associated with lower incidences of death, heart failure admission, and stroke/SE in real-world AF patients with heart failure, supporting the role of AF ablation in heart failure.

## Supplementary Information

Supplementary Information.
