# Supplementary material for: Reduction of mortality by catheter ablation in real-world atrial fibrillation patients with heart failure
Source: Sci Rep. 2021 Feb 25;11:4694. doi: 10.1038/s41598-021-84256-z (PMC7907229; doi:10.1038/s41598-021-84256-z)
Supplement: Supplementary file 1 — Supplementary Information. [file 41598_2021_84256_MOESM1_ESM.pdf]

Supplementary Materials

Reduction of mortality by catheter ablation in real-world atrial fibrillation patients with heart failure

Pil-Sung Yang[1,†], Daehoon Kim[2,†], Jung-Hoon Sung[1], Eunsun Jang[2], Hee Tae Yu[2], Tae-Hoon Kim[2], Jae-Sun Uhm[2], Jong-Youn Kim[2], Hui-Nam Pak[2], Moon-Hyoung Lee[2], and Boyoung Joung[2,*]

[1]Department of Cardiology, CHA Bundang Medical Center, CHA University, Seongnam, Republic of Korea

[2]Division of Cardiology, Department of Internal Medicine, Yonsei University College of Medicine, Seoul, Republic of Korea

†The first two authors contributed equally to this work

*Corresponding author

Address for correspondence:

Boyoung Joung, MD, PhD

50-1 Yonsei-ro, Seodaemun-gu, Seoul 03722, Republic of Korea

Phone: +82-2-2228-8460, Fax: +82-2-393-2041, E-mail: cby6908@yuhs.ac

Supplementary Table 1. Definitions and ICD-10 codes used for defining the comorbidities and clinical outcomes.

	Definitions	ICD-10 codes or conditions
**Comorbidities**		
Atrial fibrillation[1-3]	Defined from diagnosis*	ICD-10: I48
Heart failure[1-3]	Defined from diagnosis*	ICD-10: I11.0, I50, I97.1
Hypertension[1-3]	Defined from diagnosis*	ICD-10: I10, I11, I12, I13, I15 and antihypertensive medication
Diabetes mellitus[1-3]	Defined from diagnosis* plus treatment	ICD-10: E10, E11, E12, E13, E14<br>Treatment: all kinds of oral antidiabetics and insulin.
Dyslipidemia[1-3]	Defined from diagnosis*	ICD-10: E78
Ischemic stroke[1-3]	Defined from diagnosis*	ICD-10: I63, I64
Transient ischemic attack[1-3]	Defined from diagnosis*	ICD-10: G45
Hemorrhagic stroke[1-3]	Defined from diagnosis*	ICD-10: I60, I61, I62
Myocardial infarction[4]	Defined from diagnosis*	ICD-10: I21, I22, I25.2
Peripheral arterial disease[1-3]	Defined from diagnosis*	ICD-10: I70.0, I70.1, I70.2, I70.8, I70.9
Chronic kidney disease[1-3]	Defined from eGFR or diagnosis* (if laboratory value was not available, diagnosis code was used)	eGFR <60mL/min per 1.73 m^2<br>ICD-10: N18, N19
End-stage renal disease[5]	Defined from national registry for severe illness.	Patients with end-stage renal disease undergoing chronic dialysis or received a kidney transplant.
Hypertrophic cardiomyopathy[6]	Defined from at least one records of either inpatient or outpatient diagnoses	ICD-10: I42.1, I42.2
Sleep apnea	Defined from diagnosis*	ICD-10: G47.3
Proteinuria	Defined from laboratory data (if laboratory value was not available, diagnosis code was used)	Urine dipstick proteinuria 1+ or higher (ICD-10: N06, N391, N392, R80)
Osteoporosis[7]	Defined from diagnosis*	ICD-10: M80, M81, M82 (except M82.0)
Hyperthyroidism	Defined from diagnosis*	ICD-10: E05
Hypothyroidism	Defined from diagnosis*	ICD-10: E03
Chronic Liver disease	Defined from diagnosis of chronic liver disease, cirrhosis, and hepatitis	ICD-10: B18, K70, K71, K72, K73, K74, K76.1

Chronic obstructive pulmonary disease[8]	Defined from diagnosis* plus treatment	ICD-10: J42, J43(except J43.0), J44<br>Treatment: SABA, SAMA, LABA, LAMA, ICS, ICS+LABA, or methylxanthine (>1 months).
Malignancy	Defined from diagnoses of cancer (non-benign)	ICD-10: C00-C97
**Clinical outcomes**		
Ischemic stroke[1-3]	Defined from any discharge diagnoses with concomitant imaging studies	ICD-10: I63, I64
Systemic embolism[1-3]	Defined from admission diagnosis or related death	ICD-10: I74, N280 (including renal infarction)
Heart failure admission	Defined from admission diagnosis (including only main and first sub-diagnosis)	ICD-10: I11.0, I50, I97.1
Sudden cardiac arrest[9]	Defined from admission diagnosis or related death†	I46, I49.0

*To ensure accuracy, comorbidities were established based on one inpatient or two outpatient records of ICD-10 codes in the database.

†To avoid erroneous inclusion of the patients with non-cardiac arrest, we excluded the patient with sudden arrest diagnosis accomp anied by respiratory arrest (R09.0, R09.2), gastrointestinal bleeding (I85.0, K25.0, K25.4, K26.0, K26.4, K27.0, K27.4, K92.0-K92. 2), brain hemorrhage (I60.x-I62.x, S06.4-S06.6), septic shock (A41.9, R57.2), pregnancy and delivery (O00-O99), diabetic ketoacido sis (E14.1), anaphylaxis (T78.2), and accidents including suicide (T71, T75.1, T36-T65, V80-V89, W76.x, X60-X84).

eGFR, estimated glomerular filtration rate; ICD-10, International Classification of Diseases-10th Revision.

All covariates were validated in cited references.

Supplementary Table 2. Preprocedural factors associated with a likelihood of undergoing catheter ablation.

	Multivariable adjustment	
	**OR (95% CI)**	**p-value**
**Demographics**		
Age (per 10-year increase)	0.65 (0.61-0.68)	<0.001
Male	0.46 (0.41-0.53)	<0.001
High economic status	1.67 (1.55-1.80)	<0.001
AF duration (per 1-year increase)	1.04 (1.03-1.06)	<0.001
**Risk score** (per 1 increase)		
CHA_2DS_2-VASc score	0.39 (0.36-0.43)	<0.001
mHAS-BLED score*	1.60 (1.49-1.72)	<0.001
Charlson comorbidity index	1.04 (1.02-1.07)	0.001
Hospital Frailty Risk Categories	0.61 (0.57-0.67)	<0.001
**Comorbidities**		
Diabetes mellitus	1.47 (1.28-1.70)	<0.001
Ischemic stroke or TIA	3.34 (2.79-3.99)	<0.001
Hemorrhagic stroke	0.63 (0.47-0.84)	0.002
Myocardial infarction	1.51 (1.33-1.72)	<0.001
Peripheral artery disease	2.12 (1.87-2.42)	<0.001
Chronic kidney disease	0.53 (0.45-0.64)	<0.001
hyperthyroidism	1.14 (1.04-1.25)	0.007
Hypothyroidism	1.43 (1.29-1.58)	<0.001
Malignancy	1.10 (0.99-1.23)	0.077
COPD	0.89 (0.82-0.98)	0.014
Liver disease	0.76 (0.68-0.84)	<0.001
Hypertrophic cardiomyopathy	0.85 (0.70-1.03)	0.099
History of bleeding	1.38 (1.27-1.51)	<0.001

Venous thromboembolism	0.55 (0.48-0.63)	<0.001
Osteoporosis	1.42 (1.28-1.58)	<0.001

*Modified (m) HASBLED = hypertension, 1 point; >65 years old, 1 point; stroke history, 1 point; bleeding history or predisposition, 1 point; liable international normalized ratio, not assessed; ethanol or drug abuse, 1 point; drug predisposing to bleeding, 1 point.

AF, atrial fibrillation; COPD, Chronic obstructive pulmonary disease; OR, odds ratio; CI, confidence interval; TIA, transient ischemic attack.

Supplementary Table 3. Baseline characteristics of ablated and AAD treated patients before and after propensity score weighting.

	Ablation (N=3,173)	AAD treated (N=7,976)	SMD	Ablation (N=3,173)	AAD treated (N=7,976)	SMD
**Demographic**						
Age, years	60 (53, 67)	69 (60, 75)	74.6%	65 (57, 73)	65 (56, 72)	3.7%
<65 years	67.4%	36.1%	65.8%	46.8%	49.1%	4.5%
65-75 years	26.3%	32.5%	13.8%	29.4%	29.6%	0.5%
≥ 75 years	4.8%	27.7%	65.2%	20.7%	18.6%	5.3%
Male	71.5%	60.4%	23.7%	66.0%	64.7%	2.7%
High income status	51.7%	42.5%	18.4%	49.3%	45.2%	8.1%
AF duration, months	34.9 (11.9, 66.8)	23.4 (5.0, 49.9)	26.2%	21.2 (5.5, 51.2)	23.0 (3.9, 52.2)	4.9%
**Risk scores**						
CHA_2DS_2-VASc score	3.0 (2.0, 5.0)	5.0 (3.0, 6.0)	65.8%	4.0 (3.0, 6.0)	4.0 (3.0, 6.0)	5.1%
mHAS-BLED score*	3.0 (2.0, 4.0)	3.0 (2.0, 4.0)	32.2%	1.6 (1.0, 2.9)	3.0 (2.0, 3.8)	6.6%
Charlson comorbidity index	4.0 (3.0, 6.0)	5.0 (3.0, 8.0)	36.2%	5.0 (3.0, 7.0)	5.0 (3.0, 7.0)	1.3%
Hospital frailty risk score	1.8 (0.0, 4.8)	2.9 (0.0, 7.9)	57.9%	2.6 (0.5, 6.1)	2.0 (0.0, 6.2)	4.4%
**Comorbidities**						
Heart failure	100.0%	100.0%	<0.001	100.0%	100.0%	<0.001
Hypertension	91.3%	94.0%	10.6%	92.9%	91.7%	4.5%
Diabetes	17.8%	29.9%	28.7%	25.9%	25.0%	2.2%
Dyslipidemia	88.5%	81.7%	19.2%	84.3%	83.0%	3.6%
Ischemic stroke	19.9%	34.3%	32.8%	30.4%	27.9%	5.4%
TIA	10.1%	11.1%	3.2%	10.2%	10.6%	1.5%
Hemorrhagic stroke	1.5%	3.1%	11.0%	3.9%	2.5%	8.2%
Myocardial infarction	13.6%	19.9%	17.1%	16.8%	17.2%	0.9%
Peripheral arterial disease	14.2%	17.3%	8.5%	14.8%	15.9%	3.1%
Chronic kidney disease	5.9%	9.4%	13.1%	10.5%	8.0%	8.6%
End stage renal disease	0.9%	1.3%	4.6%	1.4%	1.2%	1.5%
Proteinuria	5.9%	6.9%	4.3%	6.6%	6.5%	0.5%
Hyperthyroidism	23.2%	17.9%	13.0%	18.8%	19.1%	0.8%
Hypothyroidism	20.6%	15.0%	14.7%	14.7%	16.0%	3.6%
Malignancy	21.9%	22.9%	2.4%	19.7%	22.0%	5.6%

COPD	26.2%	37.9%	25.3%	35.5%	32.9%	5.6%
Liver disease	49.4%	44.1%	10.5%	44.6%	45.4%	1.5%
Hypertrophic cardiomyopathy	3.3%	4.3%	5.4%	3.9%	4.0%	0.2%
History of bleeding	33.6%	33.8%	0.5%	35.2%	33.3%	4.0%
Osteoporosis	20.9%	31.1%	23.3%	25.4%	26.9%	3.4%
Sleep apnea	2.1%	0.6%	13.0%	1.4%	1.1%	2.8%
Heart failure admission	7.5%	26.2%	51.8%	20.4%	20.2%	0.5%
**Medication (Treatment)**	0.0%	0.0%		0.0%	0.0%	
OAC	68.2%	75.8%	17.1%	61.8%	66.2%	9.2%
Antiplatelet agents	79.8%	70.0%	22.6%	70.7%	71.5%	1.7%
ACE-inhibitor/ARB	64.2%	73.1%	19.3%	70.5%	68.2%	5.1%
Diuretics	53.7%	75.2%	46.0%	67.9%	66.2%	3.7%
K sparing diuretics	17.5%	33.3%	37.0%	27.7%	26.5%	2.6%
Statin	46.9%	45.0%	3.8%	46.0%	44.2%	3.5%
Beta blocker	77.0%	66.7%	22.9%	68.7%	68.1%	1.4%
Dihydropyridine CCB	34.7%	42.2%	15.6%	41.6%	39.1%	5.2%
Nondihydropyridine CCB	27.2%	20.8%	14.9%	21.5%	21.9%	0.9%
Digoxin	23.5%	42.7%	41.6%	35.1%	35.3%	0.5%

Values are presented as median (Q1, Q3, quartiles [25th and 75th percentiles]) or %. *Modified HAS-BLED=hypertension, 1 point: >65 years old, 1 point: stroke history, 1 point: bleeding history or predisposition, 1 point: liable international normalized ratio, not assessed: ethanol or drug abuse, 1 point: drug predisposing to bleeding, 1 point.

AAD, antiarrhythmic drug; ACE, angiotensin converting enzyme; AF, atrial fibrillation; ARB, angiotensin II receptor blocker; CCB, calcium channel blocker; COPD, chronic obstructive pulmonary disease; OAC, oral anticoagulant; SMD, standardized mean difference; TIA, transient ischemic attack.

Supplementary Table 4. Baseline characteristics of ablated and rate control only patients before and after propensity score weighting.

	Ablation (N=3,173)	Rate control only (N=5,065)	SMD	Ablation (N=3,173)	Rate control only (N=5,065)	SMD
**Demographic**						
Age, years	60 (53, 67)	70 (63, 77)	91.0%	65 (56, 73)	64 (56, 72)	1.8%
<65 years	67.4%	30.4%	79.6%	48.8%	50.2%	2.8%
65-75 years	26.3%	33.1%	15.1%	28.9%	28.9%	<0.001
≥ 75 years	4.8%	32.7%	76.5%	19.8%	18.4%	3.6%
Male	71.5%	57.1%	30.4%	65.7%	64.2%	3.3%
High income status	51.7%	42.1%	19.2%	49.3%	46.1%	6.5%
AF duration, months	34.9 (11.9, 66.8)	24.3 (4.1, 39.2)	34.2%	20.1 (5.1, 49.7)	24.2 (3.0, 47.6)	5.7%
**Risk scores**						
CHA_2DS_2-VASc score	2.0 (1.0, 3.0)	2.0 (1.0, 4.0)	81.0%	4.0 (3.0, 6.0)	4.0 (3.0, 6.0)	2.0%
mHAS-BLED score*	2.0 (2.0, 3.0)	2.0 (1.0, 3.0)	40.5%	1.6 (1.1, 2.8)	2.8 (2.0, 3.6)	5.9%
Charlson comorbidity index	3.0 (2.0, 5.0)	3.0 (1.0, 5.0)	41.1%	4.0 (3.0, 7.0)	5.0 (3.0, 7.0)	0.8%
Hospital frailty risk score	1.1 (0.0, 3.4)	0.1 (0.0, 2.9)	71.0%	2.5 (0.4, 6.0)	1.8 (0.0, 6.3)	8.7%
**Comorbidities**						
Heart failure	100.0%	100.0%	<0.001	100.0%	100.0%	<0.001
Hypertension	91.3%	95.4%	16.8%	92.8%	91.8%	3.8%
Diabetes	17.8%	32.0%	33.3%	25.0%	24.5%	1.1%
Dyslipidemia	88.5%	77.6%	29.5%	82.1%	81.3%	2.3%
Ischemic stroke	19.9%	38.3%	41.3%	29.2%	27.9%	3.0%
TIA	10.1%	11.1%	3.4%	9.9%	10.6%	2.1%
Hemorrhagic stroke	1.5%	4.0%	15.5%	3.9%	2.7%	6.7%
Myocardial infarction	13.6%	22.4%	23.2%	16.8%	17.5%	2.1%
Peripheral arterial disease	14.2%	18.7%	12.2%	14.8%	16.4%	4.3%
Chronic kidney disease	5.9%	11.0%	18.2%	10.2%	8.4%	6.3%
End stage renal disease	0.9%	1.7%	7.4%	1.4%	1.4%	0.4%
Proteinuria	5.9%	6.1%	0.8%	6.2%	6.0%	0.8%
Hyperthyroidism	23.2%	16.6%	16.5%	18.0%	18.7%	2.0%
Hypothyroidism	20.6%	14.3%	16.6%	15.0%	15.9%	2.5%

Malignancy	21.9%	22.5%	1.5%	19.6%	21.7%	5.2%
COPD	26.2%	39.3%	28.3%	34.2%	32.0%	4.7%
Liver disease	49.4%	41.9%	15.1%	44.2%	44.7%	1.2%
Hypertrophic cardiomyopathy	3.3%	3.4%	0.9%	3.4%	3.4%	0.2%
History of bleeding	33.6%	33.4%	0.4%	34.7%	33.1%	3.2%
Osteoporosis	20.9%	31.1%	23.3%	24.4%	26.0%	3.6%
Sleep apnea	2.1%	0.3%	16.4%	1.2%	1.0%	2.4%
Heart failure admission	7.5%	27.6%	54.9%	19.2%	19.1%	0.3%
**Medication (Treatment)**	0.0%	0.0%		0.0%	0.0%	
OAC	68.2%	77.5%	21.1%	57.2%	63.8%	13.6%
Antiplatelet agents	79.8%	70.9%	20.7%	71.7%	72.4%	1.6%
ACE-inhibitor/ARB	64.2%	75.1%	23.8%	68.3%	67.5%	1.7%
Diuretics	53.7%	77.7%	52.1%	65.0%	64.4%	1.1%
K sparing diuretics	17.5%	35.8%	42.4%	25.4%	25.7%	0.5%
Statin	46.9%	40.0%	13.9%	42.8%	41.3%	2.9%
Beta blocker	77.0%	62.7%	31.4%	66.0%	66.4%	0.7%
Dihydropyridine CCB	34.7%	42.8%	16.8%	40.2%	38.3%	3.9%
Nondihydropyridine CCB	27.2%	19.2%	19.0%	21.2%	21.5%	0.9%
Digoxin	23.5%	47.9%	52.5%	34.0%	35.5%	3.2%

Values are presented as median (Q1, Q3, quartiles [25th and 75th percentiles]) or %.

Abbreviations are same as Supplementary Table 3.

Supplementary Table 5. Risk of clinical outcomes in 1:1 propensity score matched ablated and different control patients.

	Number of events	Person years	Event rate (100 PYs)	Number of events	Person years	Event rate (100 PYs)	Absolute difference in event rate (95% CI)	Adjusted hazard ratio (95% CI) *	p-value
Ablation vs. Medical Therapy									
	Medical Therapy (N=2,551)			Ablation (N=2,551)					
All-cause death	448	16,625	2.7	120	11,049	1.1	1.6 (1.3-2.0)	0.38 (0.31-0.47)	<0.001
Cardiovascular death	202	16,625	1.2	45	11,049	0.4	0.8 (0.6-1.0)	0.29 (0.21-0.41)	<0.001
Heart failure	477	15,214	3.1	131	10,245	1.3	1.9 (1.5-2.2)	0.41 (0.33-0.49)	<0.001
Stroke/SE	391	15,253	2.6	132	10,493	1.3	1.3 (1.0-1.7)	0.49 (0.40-0.59)	<0.001
Sudden cardiac death	106	16,466	0.6	28	11,007	0.3	0.4 (0.2-0.6)	0.37 (0.24-0.57)	<0.001
Ablation vs. AAD treated									
	AAD treated (N=2,365)			Ablation (N=2,365)					
All-cause death	384	14,779	2.6	117	10,256	1.1	1.5 (1.1-1.8)	0.41 (0.34-0.51)	<0.001
Cardiovascular death	161	14,779	1.1	45	10,256	0.4	0.7 (0.4-0.9)	0.37 (0.27-0.52)	<0.001
Heart failure	436	13,399	3.3	125	9,460	1.3	1.9 (1.5-2.3)	0.41 (0.33-0.50)	<0.001
Stroke/SE	330	13,697	2.4	124	9,738	1.3	1.1 (0.8-1.5)	0.53 (0.43-0.65)	<0.001
Sudden cardiac death	80	14,661	0.5	30	10,204	0.3	0.3 (0.1-0.4)	0.53 (0.35-0.82)	0.004
Ablation vs. Rate control only									
	Rate control only (N=1,720)			Ablation (N=1,720)					
All-cause death	459	14,802	3.1	329	11,331	2.9	0.2 (-0.2~0.6)	0.59 (0.45-0.77)	<0.001
Cardiovascular death	196	12,274	1.6	39	7,628	0.5	1.1 (0.8-1.4)	0.28 (0.19-0.40)	<0.001
Heart failure	423	13,452	3.1	232	10,333	2.2	0.9 (0.5-1.3)	0.39 (0.32-0.48)	<0.001
Stroke/SE	432	13,167	3.3	260	10,447	2.5	0.8 (0.4-1.2)	0.33 (0.26-0.41)	<0.001
Sudden cardiac death	70	12,173	0.6	24	7,586	0.3	0.3 (0.1-0.5)	0.51 (0.32-0.82)	0.005

*Adjusted for age, sex, income, AF duration, CHA_2DS_2-VASc score, modified HAS-BLED score, hospital frailty risk score, Charlson comorbidity index, hypertension, diabetes, ischemic stroke/TIA, myocardial infarction, peripheral arterial disease, hypertrophic cardiomyopathy,

chronic kidney disease, end stage renal disease, liver disease, malignancy, hyperthyroidism, hypothyroidism, venous thromboembolism, COPD, intracranial bleeding, previous cardioversion, history of bleeding, baseline use of warfarin, non-vitamin K antagonist oral anticoagulant, aspirin, clopidogrel, beta-blocker, ACE-inhibitor/ARB, dihydropyridine/nondihydropyridine CCB, statin, diuretics, and digoxin, and OAC coverage rate of time at risk.

AAD, antiarrhythmic drug; CI, confidence interval; PYs, person-years; SE, systemic embolism. Other abbreviations are same as Supplementary Table 3.

Supplementary Table 6. Risk of clinical outcomes in propensity score weighted ablated HF patients with AF and those without AF.

	Number of events	Person years	Event rate (/100 PYs)	Number of events	Person years	Event rate (/100 PYs)	Absolute difference in event rate (95% CI)	Hazard ratio (95% CI)	p-value
Ablation vs. Patients without AF									
	No AF (N= 12,663)			Ablation (N= 3,173)					
All-cause death	1,357	79,624	1.70	397	25,105	1.58	0.12 (-0.06 ~ 0.31)	1.45 (0.52-4.06)	0.482
Heart failure	530	78,053	0.68	176	23,813	0.74	-0.06 (-0.18 ~ 0.06)	1.34 (0.39-4.61)	0.648
Stroke/SE	774	77,277	1.00	286	23,950	1.19	-0.19 (-0.34 ~ -0.04)	1.00 (0.45-2.25)	0.998

AF, atrial fibrillation; CI, confidence interval; HF, heart failure; PYs, person-years; SE, systemic embolism.

Supplementary Table 7. Risk of falsification endpoints in propensity score weighted or 1:1 propensity score matched ablated and different control patients.

	**Propensity score weighted**		**1:1 propensity score matched**	
	**HR (95% CI)**	**p-value**	**HR (95% CI)**	**p-value**
Urinary tract infection				
Ablation vs. Medical Therapy	0.94 (0.80-1.09)	0.393	0.98 (0.87-1.11)	0.778
Ablation vs. AAD treated	0.95 (0.83-1.08)	0.436	0.94 (0.84-1.06)	0.320
Ablation vs. Rate control only	0.94 (0.82-1.08)	0.371	0.98 (0.86-1.13)	0.817
Influenza infection				
Ablation vs. Medical Therapy	1.49 (0.99-2.23)	0.056	1.36 (0.96-1.93)	0.082
Ablation vs. AAD treated	1.43 (0.97-2.09)	0.068	1.25 (0.87-1.79)	0.231
Ablation vs. Rate control only	1.37 (0.92-2.05)	0.118	1.13 (0.78-1.63)	0.519
Varicella-zoster virus infection				
Ablation vs. Medical Therapy	1.15 (0.95-1.40)	0.159	1.19 (0.99-1.43)	0.067
Ablation vs. AAD treated	1.10 (0.92-1.32)	0.287	1.04 (0.86-1.25)	0.673
Ablation vs. Rate control only	1.20 (0.99-1.46)	0.060	1.20 (0.97-1.48)	0.090
Fall accident				
Ablation vs. Medical Therapy	0.65 (0.24-1.76)	0.391	1.12 (0.21-5.83)	0.894
Ablation vs. AAD treated	0.69 (0.25-1.93)	0.478	1.61 (0.34-7.59)	0.549
Ablation vs. Rate control only	0.99 (0.32-3.06)	0.989	1.32 (0.22-7.74)	0.761

HR, hazard ratio; CI, confidence interval.

Supplementary Figure 1. Validation of the definitions for detecting AF recurrence, using the results of ECG/Holter monitoring with a total of 212 patients from a tertiary cardiovascular center.

(A) AF recurrence was defined by cardioversion or redo-ablation beyond a 3 months blanking period.

	**Proven recur (+) (N=87)**	**Proven recur (-) (N=125)**	
**AF recurrence (N=43)**	42	1	**PPV: 97.7%**
**No AF recurrence (N=169)**	45	124	**NPV: 73.4%**
	**Sensitivity: 48.3%**	**Specificity: 99.2%**	

(B) Kaplan–Meier curve of time to defined AF recurrence (cardioversion or redo-ablation) in ablated patients.

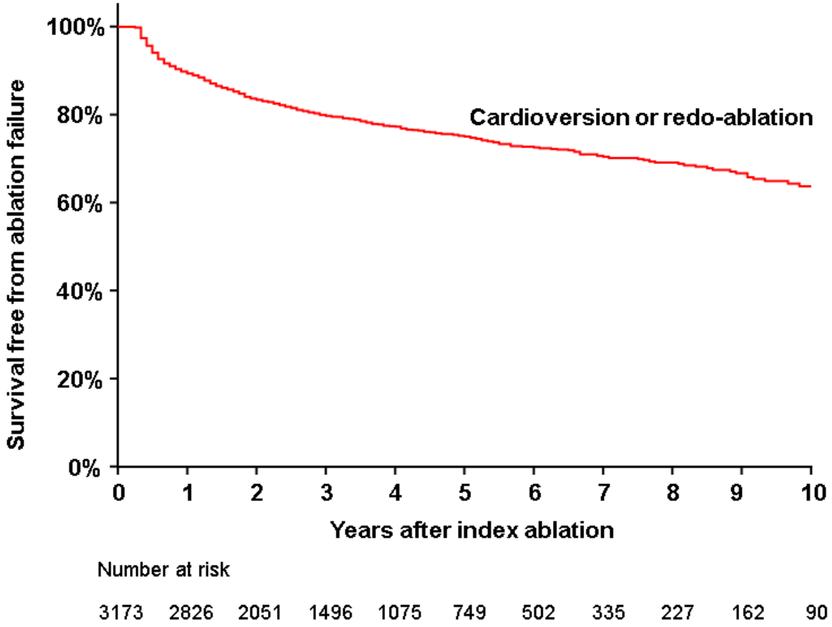

References

1. Kim D, Yang PS, Jang E, et al. Increasing trends in hospital care burden of atrial fibrillation in Korea, 2006 through 2015. Heart 2018.
2. Lee H, Kim TH, Baek YS, et al. The Trends of Atrial Fibrillation-Related Hospital Visit and Cost, Treatment Pattern and Mortality in Korea: 10-Year Nationwide Sample Cohort Data. Korean Circ J 2017;47:56-64.
3. Kim TH, Yang PS, Kim D, et al. CHA2DS2-VASc Score for Identifying Truly Low-Risk Atrial Fibrillation for Stroke: A Korean Nationwide Cohort Study. Stroke 2017;48:2984-2990.
4. Lee HY, Yang PS, Kim TH, et al. Atrial fibrillation and the risk of myocardial infarction: a nation-wide propensity-matched study. Sci Rep 2017;7:12716.
5. Seong SC, Kim YY, Park SK, et al. Cohort profile: the National Health Insurance Service-National Health Screening Cohort (NHIS-HEALS) in Korea. BMJ Open 2017;7:e016640.
6. Jung H, Yang PS, Sung JH,et al. Hypertrophic Cardiomyopathy in patients with Atrial Fibrillation: Prevalence and stroke risks in a nationwide cohort study. Thrombosis and Haemostasis. 2019 Feb;119(2):285-293.
7. Kim D, Yang PS, Kim TH, et al. Effect of Atrial Fibrillation on the Incidence and Outcome of Osteoporotic Fracture - A Nationwide Population-Based Study. Circulation journal. 2018 Jul 25;82(8):1999-2006.
8. Song S, Yang PS, Kim TH, et al. Relation of Chronic Obstructive Pulmonary Disease to Cardiovascular Disease in the General Population. Am J Cardiol 2017;120:1399-1404.
9. Kim IJ, Yang PS, Kim TH, et al. The relationship between Anemia and the Risk of Sudden Cardiac Arrest: A Nationwide Cohort Study in South Korea. Circulation Journal. 2018 Nov 24;82(12):2962-2969.
